# The Association of Dry Eye Disease with Functional Visual Acuity and Quality of Life

**DOI:** 10.3390/jcm12237484

**Published:** 2023-12-04

**Authors:** Lydia Hui-Peng Tan, Louis Tong

**Affiliations:** 1 Yong Loo Lin School of Medicine, National University of Singapore, Singapore 118417, Singapore; lydia_huipengtan@u.nus.edu; 2 Corneal and External Diseases Department, Singapore National Eye Centre, Singapore 168751, Singapore; 3 Ocular Surface Group, Singapore Eye Research Institute, Singapore 169856, Singapore; 4 Eye Academic Clinical Program, Duke-NUS Medical School, Singapore 169857, Singapore

**Keywords:** ocular surface, cornea, dry eye disease, meibomian gland dysfunction, tear disorders, functional visual acuity, case-controlled study

## Abstract

Background: Dry eye disease (DED) is a common chronic condition with increasing prevalence. Standard discriminative visual acuity is not reflective of real-world visual function, as patients can achieve normal acuities by blinking. Methods: Participants recruited from a tertiary referral eye centre were divided into two groups—Severe DED (with significant, central staining) and Mild DED (absence of such staining). Functional Visual Acuity (FVA) in both groups was assessed using the DryeyeKT mobile application and Impact of Vision Impairment (IVI) questionnaire to assess quality of life (QOL). Results: Among the 78 participants (74.4% women), 30 (38.5%) had Severe DED and 48 (61.5%) Mild DED. In women, Severe DED produced a significantly worse FVA of 0.53 ± 0.20 vs. 0.73 ± 0.30 in the Mild DED group (*p* = 0.006). FVA decreased with increasing age, showing a significant inverse correlation (r = −0.55). A poorer FVA ≤ 0.6 was seen in older patients (68.2 years ± 7.68) vs. an FVA > 0.6 in younger patients (58.9 years ± 10.7), *p* < 0.001. When adjusting for age, FVA was still 0.107 lower in the Severe DED group, *p* = 0.003. There was significant difficulty in performing specific daily activities in the Severe DED group, after adjusting for age, gender and FVA. Conclusions: FVA is reduced in severe DED and older people. Severe DED significantly impacts certain aspects of QOL. However, no significant relationship was found between FVA and QOL. FVA is not the only reason for the compromise of health-related QOL in severe dry eye.

## 1. Introduction

Dry eye disease (DED) is a common chronic condition [1,2,3,4,5] that significantly affects quality of life (QOL) [6,7,8], often presenting with symptoms of grittiness, burning and foreign body sensation. It causes a great impairment of functional visual acuity [9], limiting vision-related activities in the day-to-day life of patients, such as reading and driving [10,11]. Epidemiological studies suggest a prevalence rate ranging from 5 to 50% in different populations, and being Asian is one of the risk factors for the development of DED [2]. More notably, there is an increasing prevalence of DED as society continues to age [12,13].

The multifactorial aetiology of DED can be simplified to two main mechanisms—decreased tear production and/or increased evaporative loss [14]. The tear film consists of three main layers—the inner mucin layer, middle aqueous layer and outer lipid layer—which provide a barrier to minimise tear evaporation from the ocular surface [15].

The diagnosis and severity of DED are often assessed through thorough history taking and symptom questionnaires in combination with clinical signs and ocular examinations [16,17]. However, there is currently no objective method that is routinely used to determine visual function in dry eye.

The reason for poor tear function in dry eye is that poor tear stability [18] induces an uneven tear film between blinks, inducing optical aberrations [19]. Tear dysfunction is also a major cause of superficial corneal epithelial disease [20]. In cases of dry eye with cornea epitheliopathy (shown by fluorescein staining), the ocular surface as an optical medium may also scatter light due to lack of homogeneity [20,21,22]. Conventional visual acuity screening is significantly limited in dry eye patients, as they can achieve near-normal acuity levels simply by increasing the frequency of blinking to compensate for an inadequate tear film during the examination [23]. Therefore, a more dynamic form of vision assessment that simulates real-world visual tasks is the preferred approach.

Over the years, various mobile applications have been developed as a self-screening tool for patients to estimate their probability of having DED [24,25]. The mobile application “You Can Know Whether You Have Dry Eye in a Minute” uses dynamic testing methods to test for functional visual acuity (FVA), coupled with validated DED symptom questionnaires, while allowing subjects to blink naturally during the measurement period. A study revealed that tear film breakup time (TBUT) was significantly shorter among subjects with DED identified by the application [25].

The objective of this study was firstly to determine if FVA (performed via the smartphone application) was reduced in severe dry eye and its correlation to QOL. Secondly, we aimed to evaluate the correlation between this FVA and other clinical parameters of DED.

## 2. Materials and Methods

### 2.1. Study Population

A prospective cross-sectional, comparative study was conducted involving 78 participants recruited from the Singapore National Eye Center. Thirty participants were recruited to the severe dry eye (Severe DED) group and 48 participants were recruited to the comparison mild dry eye (Mild DED) group. Diagnosis of dry eye was made by referring physicians prior to coming to our clinic. These patients all demonstrated the presence of dry eye symptoms, with either a TBUT < 5 or the presence of corneal staining. 

Participants recruited to the Severe DED group had central corneal fluorescein staining in both eyes, while those recruited to the Mild DED group did not have central corneal fluorescein staining in any eye. Corneal fluorescein staining was performed using the Oculus Keratograph 5M [26] and scored in 5 corneal zones—superior, inferior, nasal, temporal and central. The inclusion criteria for the study participants comprised an age of 21 years or older during the time of study and a narrow range of Visual Acuity (VA) of between 6/6 and 6/9.5. 

Participants meeting any of the following exclusion criteria were excluded from this study. These include participants who were unsuitable or unable to put their chin on the chin rest for the Oculus Keratograph 5M (Wetzlar, Germany) and slit lamp as well as participants who were pregnant, lactating or planning a pregnancy [27]. Pathological conditions in elderly eyes such as age-related macular degeneration (AMD) [28] and macular oedema [29] also led to exclusion. Participants with highly asymmetrical cases between the two eyes were not recruited.

The study was conducted in accordance with the tenets in the Declaration of Helsinki that are consistent with the Good Clinical Practice and Human Biomedical Research Act, Singapore. Written informed consent was obtained from all participants. The protocol was approved by the Singhealth Centralised Institutional Review Board (CIRB).

### 2.2. Study Outcomes

Participants’ characteristics, such as age and gender, were collected. A medical history was obtained, and previous dry eye treatment (if any) was recorded. Risk factors and potential causes of DED were identified, including history of allergies [30], history of dry mouth, history of contact lens wear, history of systemic diseases including autoimmune disorders and thyroid diseases [16], and history of medications [31] used within 1 month. A history of any previous ocular surgery was also recorded [31].

The primary outcomes were FVA in the severe dry eye group compared to the comparison group and QOL. The secondary outcomes were the clinical parameters of DED, including standard patient evaluation of eye dryness (SPEED), non-invasive tear break-up time (NIBUT), meibomian gland signs and Schirmer I readings.

### 2.3. Study Procedures

#### 2.3.1. FVA

Functional Visual Acuity refers to an individual’s performance in relation to daily activities involving visual tasks. This was evaluated using the DryeyeKT mobile application developed by Kazuo Tsubota [25]. Participants were required to hold the mobile device 60 cm away from them for the 30 s FVA testing on the application. A single landolt C was shown in the middle of the screen, and participants were required to tap on the arrow of the direction of the broken ring; the size of the landolt C was adjusted in depending on whether the participant gave the correct response. The direction of the landolt C was randomised to up, down, left or right while waiting for the participants’ response. The application generated a score ranging from 0 (absent) to 2 (perfect visual function).

#### 2.3.2. QOL Questionnaire

The Impact of Vision Impairment (IVI) questionnaire© assessing the QOL was administered in the interviewer format. The questionnaire has been validated across various ocular conditions [32,33] and various populations [34,35,36,37]. The questionnaire includes 28 questions divided into 3 domains: (i) reading and accessing information (9 items), (ii) mobility and independence (11 items), and (iii) emotional well-being (8 items). Response options used the Likert scale. 

#### 2.3.3. Tear Break-Up Time

This was assessed using a Keratograph 5M [26] (Oculus, Wetzlar, Germany). Briefly, patients blink freely while fixing on a target ahead. Once ready, patients blink twice and then refrain from further blinking. The fully automated instrument captures any break or distortion in the image of the projected rings on the cornea, and the timings are automatically recorded. Higher readings indicate more tear stability. 

#### 2.3.4. Corneal Fluorescein Staining

Corneal fluorescein staining was also performed using the Keratograph 5M and scored in 5 corneal zones as in the Brien Holden Vision Institute (BHVI) system [38], with a greater score indicating a more intense or greater area of staining. 

#### 2.3.5. Standard Patient Evaluation of Eye Dryness (SPEED)

The SPEED questionnaire© consists of 4 questions on the frequency and severity of dry eye graded on a scale of 0–3 on frequency, and grades 0–4 on severity. Scores from all sub-questions were added, and the greater the total score (0–28), the more frequent or severe the dry eye [39].

#### 2.3.6. Schirmer’s I Test

Schirmer test [40] was done with the standard 5 mm wide test strips (Clement Clarke International Ltd., Harlow, Essex, UK). The strips were positioned over the inferior temporal half of the lower lid margin in both eyes, and participants were required to close their eyes. The extent of wetting of the strips was recorded after 5 min, and strips were stored at −80 °C.

#### 2.3.7. Meibomian Gland Dysfunction (MGD) Examination

The meibomian glands were assessed by gently squeezing the lower eyelids using a device that delivers standardised pressure to the eyelids (Meibomian gland expressor, TearScience, Johnson & Johnson Vision, Milpitas, CA, USA). Textures of the expressed secretion were graded as liquid or viscous [41]. 

### 2.4. Statistical Analysis

Sample size calculation was based on the clinical outcome of the Uchino 2018 [25] study on mobile application. We assumed a mean FVA score of 0.76 ± 0.04 in Severe DED and a 10% decrease in FVA for Mild DED, as anything smaller was unlikely to be clinically significant even if it was statistically significant. Sample size was determined using calculator software https://clincalc.com/stats/samplesize.aspx (accessed on 14 November 2023).

We aimed to achieve a significance level (α) of 0.05, power of 80% and sampling ratio of 1. To account for participants lost to follow-up, we recruited more than the required number. The difference between the number of participants in both groups was due to the consecutive recruitment of participants in our clinic, where a smaller proportion of patients have Severe DED.

Statistical analysis was performed using StataCorp. 2013 (Stata Statistical Software: Release 13.1. College Station, TX, USA: StataCorp LP). Statistical significance was at a two-tailed *p*-value of 0.05. A two-tailed *t* test was used to assess the mean differences of continuous variables such as FVA, overall IVI, NIBUT, staining grade, SPEED score, Schirmer’s test score and number of liquid-expressing glands. Fisher’s exact test was used for ordinal and categorical variables. Some continuous variables were categorised into binary categories according to meaningful clinical thresholds, and their associations were re-evaluated using the Fisher’s test via a 2 × 2 table. FVA score was categorised into ≤0.6, signifying poorer vision, and >0.6, as the comparison group with better vision. An FVA of 0.6 was used as the threshold as it was between the means of both the severe DED and milder DED group.

Variables were explored by first plotting a histogram to analyse the distribution and identify any outliers. Each item of the IVI was analysed separately in addition to the total score. Multiple logistic regression models were performed, with each question of the IVI questionnaire as a dependent variable. Covariates of the models were added incrementally. The correlation between FVA and other clinical parameters in the assessment of dry eye, as well as IVI scores, was also analysed.

## 3. Results

### 3.1. Participants’ Characteristics

A total of 78 participants were analysed. Thirty (38.5%) participants were from the Severe DED group, while 48 (61.5%) participants were from the comparison group with Mild DED. (Table 1) There was similar gender distribution in both groups (*p* = 1.00), although a large preponderance were female (73.3% in the Severe DED group vs. 75% in the Mild DED group). The mean age of participants in the Severe DED group was 63.9 ± 11.2, while the mean age of participants in the Mild DED group was 62.7 ± 10.4, *p* = 0.63. No difference was observed between the ages of participants in both groups, with the majority being above 50 years (90% vs. 87.5%, *p* = 1.00). Table 2 details the Ophthalmic and medical conditions of participants in both groups. 

### 3.2. Assessment of Dry Eye

As expected, corneal fluorescein staining grade was significantly elevated in the Severe DED group compared to the Mild DED group in all quadrants for both eyes (Table 3). The inferior zone of the cornea is most commonly affected by dry eye [42,43].

However, there was no significant difference between the NIBUT scores of the two groups. The mean NIBUT in the Severe DED group and the Mild DED group was 6.89 ± 5.46 s and 9.15 ± 6.73 s, respectively (*p* = 0.13). 

SPEED questionnaire scores were slightly worse in the Severe DED group, but this was not statistically significant. Participants in the Severe DED group had a score of 8.17 ± 6.64 compared to 5.29 ± 5.92 in the Mild DED group (*p* = 0.050). 

The Schirmer’s test score was significantly higher in the Mild DED group (8.49 ± 9.80) than in the Severe DED group (3.53 ± 5.54), *p* = 0.014, for the right eye, and trended towards a higher score for the left eye as well. (Table 4) This means that there was significantly more wetting of the Schirmer test strip and hence greater tear production in the Mild DED group.

The total number of meibomian glands expressed was similar in both the Mild DED group and Severe DED group, *p* = 0.69 in the right eye and *p* = 0.58 in the left eye. However, significantly more meibum expressed was liquid in character in the Mild DED group compared to that in the Severe DED group, *p* = 0.045 in the right eye and *p* = 0.012 in the left eye (Table 5). Normal liquid meibum is clear and easily expressed, while viscous meibum which is usually associated with meibomian gland disease, can be difficult to express [44].

### 3.3. Functional Visual Acuity (FVA)

For the FVA testing, two patients (in the Severe DED group) were excluded from the analysis because the FVA mobile application was not available at the time of assessment. 

**Figure 1:**
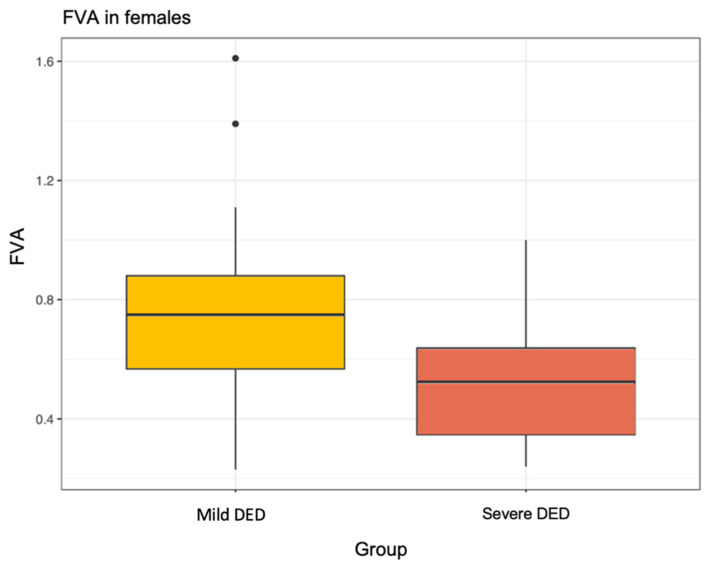
Functional visual acuity (FVA) in females.

**Figure 2:**
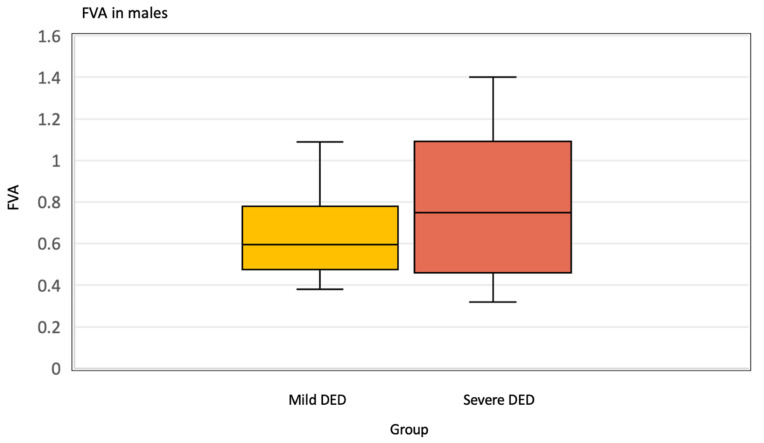
Functional visual acuity (FVA) in males.

We observed that in women, Severe DED produced a significantly worse FVA of 0.52 ± 0.20 compared to 0.73 ± 0.30 in the Mild DED group, *p* = 0.006 (Figure 1). There were not enough men in the study to show this relationship (Figure 2) (Table 6).

**Figure 3:**
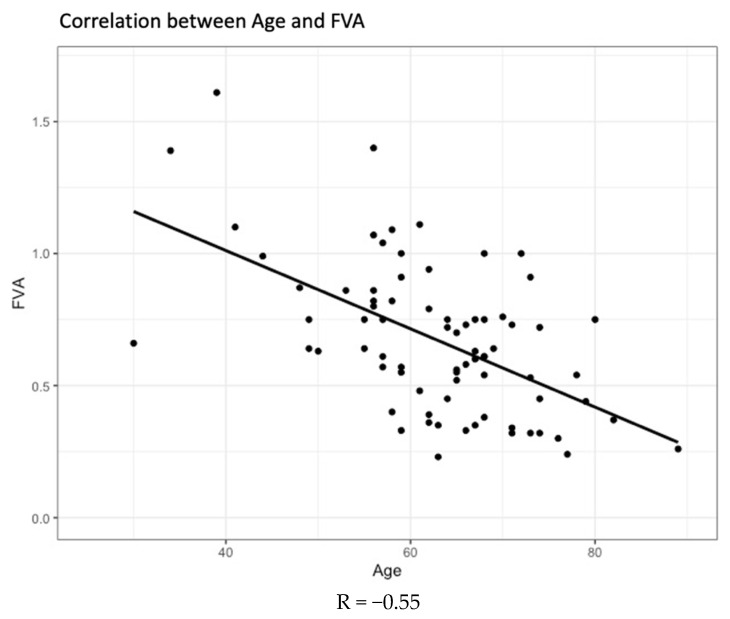
Correlation between age and functional visual acuity (FVA).

**Figure 4:**
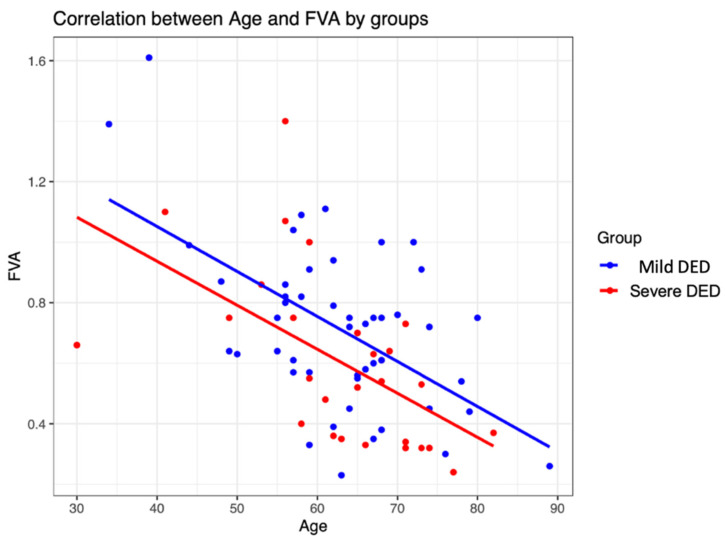
Correlation between age and functional visual acuity (FVA) by group.

In general, functional visual acuity was observed to decrease with increasing age (Figure 3), with the two factors sharing a negative correlation coefficient (r = −0.55). The same trend was observed when analysing the two groups of participants separately (Figure 4).

When adjusting for age, FVA was observed to be 0.107 lower in the Severe DED group compared to the Mild DED group, *p* = 0.003.

An FVA of 0.6 was used as the threshold, as it was between the means of both groups. A poorer FVA ≤ 0.6 was seen in older patients (68.2 years ± 7.68) compared to an FVA > 0.6 in younger patients (58.9 years ± 10.7), *p* < 0.001 (Table 7). The Schirmer test score was also significantly higher in the FVA > 0.6 group (8.78 ± 10.3 vs. 3.15 ± 3.69, *p* = 0.005). There were no significant associations found between FVA and staining grade, NIBUT, number of meibomian glands expressed or SPEED.

Severe DED did not significantly affect overall IVI (Table 8)**,** with a score of 75.2 ± 11.1 in the Severe DED group compared to 76.3 ± 13.3 in the Mild DED group (*p* = 0.70).

Q26 and Q28 were further analysed given that the results were close to a significance of *p* = 0.05. When adjusting for age, gender and FVA, participants in the Severe DED group showed significantly more concerns or worries about coping with everyday life (Table 9) and interference with life in general (Table 10).

When using an FVA of 0.6 as a cut-off, participants in both the FVA ≤ 0.6 and FVA > 0.6 groups had similar IVI scores (73.8 ± 15.9 vs. 77.0 ± 9.43, *p* = 0.28) (Table 11).

## 4. Discussion

### 4.1. Summary of Significant Findings and Comparison with Literature

Our study found that severe dry eye with severe central corneal fluorescein staining significantly impairs the FVA in DED. Patients with Severe DED performed poorer in identifying the direction of landolt C presented on the application, requiring larger fonts for near vision, even when blinking naturally during the testing. Previous studies have shown that FVA in severe DED decreased significantly when subject eyes were kept open for 10–20 s [9]. When dry eye patients compensated by blinking twice as much [45], this improved tear distribution across the cornea, reduced tear film break-up [46,47], and decreased the exposed ocular surface for tear evaporation [15,48]. This allowed patients to attain a normal best corrected visual acuity (BCVA) score on conventional visual acuity testing [49]. However, recent studies support our findings that severe DED patients benefit from dynamic testing that substantiated their subjective visual complaints [50]. Moreover, tasks requiring long periods of focus and near work are associated with a further decrease in spontaneous blinking rates [51,52,53], further contributing to the decrease in FVA in patients with severe dry eye. 

A reduction in FVA did not impair all types of visually dependent activities but nonetheless posed significant concerns and problems with coping with daily life. While activities such as driving were not included in our QOL questionnaire, previous studies have shown that DED interfered with driving [11] and posed safety concerns [54]. Work productivity was also found to be reduced, contributing to a high economic burden [55,56,57,58]. It is interesting to note that while there were no significant differences in difficulty performing the majority of the daily activities between the two groups, those with severe dry eye had significantly increased feelings of nuisance or burden and interference with life in general. This could be attributed to the varying severity of DED among the patients recruited and that most of them were still able to compensate well or had developed various coping mechanisms [52]. Nonetheless, the psychological burden [59] and mental stress [60] of coping with constant symptoms should not be negated [61].

Our findings also suggest that older age impairs FVA marginally but takes more than 10 additional years to show its effect. A poorer FVA ≤ 0.6 was seen in older patients of 68.2 years mean age compared to FVA > 0.6 in younger patients of 58.9 years mean age. Hence, the prevalence of DED is expected to increase owing to the rapidly ageing population. Various studies have shown that the symptoms and signs of dry eye are common in the older population [12,62,63,64,65,66,67,68,69]. This observation can be explained by various physiological changes in the human body brought about by senescence, such as the decrease in the number of active meibomian glands [70], gland dropout [65,71] and plugging of meibomian orifices [65]. The meibomian gland, which is responsible for the production of the superficial oily layer of the precorneal tear film [72], undergoes acinar cell atrophy and hyperkeratinization of the ductal epithelium with age [73,74], subsequently resulting in decreased tear flow [75,76].

Lastly, we were unable to prove a correlation between FVA and NIBUT as measured by the Ocular K5M. FVA is statistically well known to have a negative correlation with DED [77], and a reduction in TBUT is often used in the diagnosis of DED and assessment of its severity [78] and was even used for recruitment of patients to the severe dry eye group [25] in earlier studies. Yet, there was no significant difference between the tear film break-up time in the two groups in our study, although we found that TBUT tended to be worse in the severe dry eye group (6.89 ± 5.46 s vs. 9.15 ± 6.73 s, *p* = 0.13). Anecdotally we noticed poorly reproducible NIBUT readings in the patients with significant corneal staining, which could influence the robustness of the findings in the severe DED group.

The results of this study may provide guidance on methods for physicians to assess visual acuity–related symptoms that patients report. The use of existing mobile phone applications such as the DryeyeKT application in clinical practice could be beneficial, or newer dynamic methods of testing can be developed.

### 4.2. Strengths and Weaknesses

The main strength of our study is that we are the first to report the correlation between FVA performed via the DryeyeKT application and commonly used clinical parameters in the assessment of dry eye.

However, the study was limited by a small database with less than 100 subjects and the lack of a true control group, as participants in our comparison group had mild DED. To evaluate the normal range of FVA in specific age groups, we need to establish a population-based study enrolling thousands of subjects. Another significant limitation was the lack of checking for refractive error, which could affect FVA values. We did not perform fluorescein BUT in this study. Our findings may have selection bias, as subjects enrolled were from a single hospital in Singapore. In addition, the DryeyeKT application only assessed the differences in FVA between the two groups on near vision. Future studies could be done to study the impact of dry eye symptoms on distant vision.

## 5. Conclusions

In conclusion, dry eye disease is a multifactorial disease anticipated to be increasing in prevalence as our society ages. Dry eye disease causes a significant reduction in the functional visual acuity of patients, which is often not picked up on conventional visual acuity testing. Dynamic methods of testing specific for patients with DED are recommended, and consideration can be given to starting treatment in patients with significant loss of QOL or difficulty performing daily visually dependent activities as a result of their dry eye.

## Figures and Tables

**Table 1:** Demographic characteristics of participants in Severe DED and Mild DED groups.

	Overall	Severe DED	Mild DED	*p*-Value
Overall N (%)	78 (100)	30 (38.5)	48 (61.5)	
Gender N (%)				
Male	20 (25.6)	8 (40)	12 (60)	1.00
Female	58 (74.4)	22 (37.9)	36 (62.1)	
Age				
Mean (SD)	63.1 (10.7)	63.9 (11.2)	62.7 (10.4)	0.63
<50 years	9	3	6	1.00
≥50 years	69	27	42	

DED: dry eye disease. SD: standard deviation.

**Table 2:** Ophthalmic and medical conditions of participants in Severe DED and Mild DED groups.

	Overall (*n* = 78)	Severe DED (*n* = 30)	Mild DED (*n* = 48)	*p*-Value
Allergies (Eczema, sinusitis, childhood asthma)				
Present	23	10	13	0.62
Absent	55	20	35	
Dry mouth				
Present	20	9	11	0.60
Absent	58	21	37	
Contact lens				
Wearer	4	2	2	0.64
Non-wearer	74	28	46	
Systemic diseases				
Rheumatoid Arthritis	3	2	1	0.56
Thyroid disease	7	3	4	1.00
Sjogren’s syndrome	4	3	1	0.29
Diabetes Mellitus Type I	7	3	4	1.00
Diabetes Mellitus Type II	2	0	2	0.52
Diet-controlled or Impaired glucose tolerance	1	0	1	1.00
None of the above	58	21	37	
Medications				
Oral contraceptives	0	0	0	1.00
Antihistamine	8	1	7	0.14
Anti-hypertensive	19	7	12	1.00
Antidepressants	0	0	0	1.00
Anti-Parkinson’s	0	0	0	1.00
Lomotil	0	0	0	1.00
Immunosuppressant (Prednisolone, cyclosporine, SMF, tacrolimus)	2	1	1	1.00
None of the above	51	22	29	
History of Ocular surgery				
LASIK				
R eye	2	1	1	1.00
L eye	0	0	0	1.00
Bilateral	4	1	3	1.00
Cataract ^1^				
R eye	1	1	0	0.39
L eye	2	0	2	0.52
Bilateral	22	12	10	0.077
Others ^2^	6	3	3	0.67
Smoking	0	0	0	1.00

^1^ Cataracts are not clinically significant. ^2^ Three participants in the Severe DED group underwent laser peripheral iridotomy to both eyes, blepharoplasty to both eyes and an unspecified ocular surgery for complication of DM Type II, respectively. Three participants in the Mild DED group underwent multiple right eyelid surgery for ptosis, squint surgery and surgery for glaucoma (micropulse laser trans-scleral cyclophotocoagulation and trabeculectomy), respectively. DED: dry eye disease.

**Table 3:** Assessment of dry eye—non-invasive tear break-up time (NIBUT), corneal fluorescein staining grade and standard patient evaluation of eye dryness (SPEED) scores.

	Overall (*n* = 78)	Severe DED (*n* = 30)	Mild DED (*n* = 48)	*p*-Value
**NIBUT (%) R eye**				
<3 s n (%)	11 (14.1)	2 (18.2)	9 (81.8)	0.19
> or =3 s n (%)	67 (85.9)	28 (41.8)	39 (58.2)	
**NIBUT (%) L eye**				
<3 s n (%)	12 (15.4)	8 (66.7)	4 (33.3)	0.050
> or =3 s n (%)	66 (84.6)	22 (33.3)	44 (66.7)	
**NIBUT (s)**				
Mean (SD)	8.29 (6.35)	6.89 (5.46)	9.15 (6.73)	0.13
**Staining grade (superior)** **R eye**				
Mean (SD)	0.22 (0.66)	0.55 (0.97)	0.02 (0.14)	**<0.001**
**Staining grade (inferior)** **R eye**				
Mean (SD)	1.18 (1.41)	2.45 (1.35)	0.39 (0.67)	**<0.001**
**Staining grade (nasal)** **R eye**				
Mean (SD)	0.83 (1.32)	1.97 (1.50)	0.13 (0.38)	**<0.001**
**Staining grade (temporal)** **R eye**				
Mean (SD)	0.64 (1.11)	1.52 (1.32)	0.09 (0.41)	**<0.001**
**Staining grade (central)** **R eye**				
Mean (SD)	0.63 (1.12)	1.65 (1.27)	0 (0)	**<0.001**
**Staining grade (total)** **R eye**				
Mean (SD)	3.51 (4.88)	8.13 (5.05)	0.63 (1.07)	**<0.001**
**Staining grade (superior)** **L eye**				
Mean (SD)	0.46 (0.83)	1.03 (1.02)	0.10 (0.37)	**<0.001**
**Staining grade (inferior)** **L eye**				
Mean (SD)	1.21 (1.48)	2.57 (1.40)	0.35 (0.68)	**<0.001**
**Staining grade (nasal)** **L eye**				
Mean (SD)	1.12 (1.45)	2.55 (1.33)	0.23 (0.47)	**<0.001**
**Staining grade (temporal)** **L eye**				
Mean (SD)	0.81 (1.37)	2.05 (1.53)	0.04 (0.20)	**<0.001**
**Staining grade (central)** **L eye**				
Mean (SD)	0.79 (1.32)	2.07 (1.38)	0 (0)	**<0.001**
**Staining grade (total)** **L eye**				
Mean (SD)	4.40 (5.70)	10.3 (5.19)	0.73 (0.99)	**<0.001**
**SPEED**				
Mean (SD)	6.40 (6.32)	8.17 (6.64)	5.29 (5.92)	0.050

DED: dry eye disease. SD: standard deviation.

**Table 4:** Results of Schirmer test in both groups.

	Overall (*n* = 77)	Severe DED (*n* = 30)	Mild DED (*n* = 47 ^1^)	*p*-Value
**Schirmer reading (R eye)**				
Mean (SD)	6.56 (8.70)	3.53 (5.54)	8.49 (9.80)	**0.014**
**Schirmer reading (R eye)**				
Normal (>15 mm)	10	1	9	0.079
Low normal (11–15 mm)	6	2	4	1.00
Borderline (6–10 mm)	11	4	7	1.00
Abnormal (<6 mm)	50	23	27	0.094
**Schirmer reading (L eye)**				
Mean (SD)	6.14 (8.53)	4.1 (7.10)	7.45 (9.17)	0.093
**Schirmer reading (L eye)**				
Normal (>15 mm)	9	1	8	0.082
Low normal (10–15 mm)	5	3	2	0.37
Borderline (6–10 mm)	13	4	9	0.76
Abnormal (<6 mm)	50	22	28	0.23

^1^ Schirmer’s test was not conducted for one patient in the Mild DED group due to patient refusal. DED: dry eye disease. SD: standard deviation.

**Table 5:** Meibomian gland expression and character in Severe DED and Mild DED groups.

	Overall (*n* = 78)	Severe DED (*n* = 30)	Mild DED (*n* = 48)	*p*-Value
**Number of Meibomian glands expressed (R eye)**				
Mean (SD)	2.14 (1.86)	2.03 (1.75)	2.21 (1.93)	0.69
Median (Range)	2 (0–8)	2 (0–6)	2 (0–8)	
**Meibomian gland expression character** **(R eye)**				
Liquid	53	16	37	**0.045**
Viscous	18	10	8	0.11
Not expressible	7	4	3	0.42
**Number of Meibomian glands expressed (L eye)**				
Mean (SD)	2.76 (2.34)	2.57 (2.03)	2.88 (2.53)	0.58
Median (Range)	3 (0–10)	2 (0–8)	3 (0–10)	
**Meibomian gland expression character** **(L eye)**				
Liquid	53	15	38	**0.012**
Viscous	19	11	8	0.060
Not expressible	6	4	2	0.20

DED: dry eye disease. SD: standard deviation.

**Table 6:** Summary of FVA in Severe DED and Mild DED groups.

	Overall	Severe DED ^1^	Mild DED	*p*-Value
Mean (SD)	0.67 (0.28)	0.60 (0.28)	0.71 (0.28)	0.096
N	76	28	48	
Gender				
Male				
Mean (SD)	0.72 (0.29)	0.80 (0.37)	0.66 (0.22)	0.29
N	20	8	12	
Female				
Mean (SD)	0.66 (0.28)	0.52 (0.20)	0.73 (0.30)	**0.006**
N	56	20	36	
Age				
≤50 years				
Mean (SD)	0.96 (0.35)	0.84 (0.23)	1.02 (0.40)	0.49
N	9	3	6	
>50 years				
Mean (SD)	0.63 (0.25)	0.57 (0.28)	0.67 (0.23)	0.13
N	67	25	42	

^1^ The FVA test was not conducted for two patients in the Severe DED group. FVA: functional visual acuity. DED: dry eye disease. SD: standard deviation.

**Table 7:** Functional visual acuity (FVA) association with other clinical parameters of dry eye.

	Overall (*n* = 76)	FVA ≤ 0.6 (*n* = 32)	FVA > 0.6 (*n* = 44)	*p*-Value
**Gender N (%)**				
Male	20	8 (40)	12 (60)	1.00
Female	56	24 (42.9)	32 (57.1)	
**Age**				
Mean (SD)	62.8 (10.6)	68.2 (7.68)	58.9 (10.7)	**<0.001**
**Staining Grade (total)**				
Mean (SD)	3.67 (5.08)	4.42 (5.10)	3.13 (5.02)	0.28
**NIBUT (s)** ^1^				
Mean (SD)	8.39 (6.39)	7.26 (5.50)	9.22 (6.89)	0.19
**Schirmer** ^2^				
Mean (SD)	6.45 (8.66)	3.15 (3.69)	8.78 (10.3)	**0.005**
**Number of Meibomian glands expressed**				
Mean (SD)	2.46 (2.14)	2.17 (2.18)	2.67 (2.10)	0.32
Median (Range)	2 (0–10)	2 (0–10)	3 (0–9)	
**SPEED** ^3^				
Mean (SD)	6.43 (6.35)	6.03 (7.01)	6.73 (5.88)	0.64

^1^ NIBUT = Non-invasive tear break-up time. ^2^ The Schirmer test was not conducted for one patient in the Mild DED group. ^3^ SPEED = Standard patient evaluation of eye dryness. SD: standard deviation.

**Table 8:** Severe dry eye disease (DED) association with impact of vision impairment (IVI) questionnaire.

	Overall (*n* = 78)	Severe DED (*n* = 30)	Comparison (*n* = 48)	*p*-Value
**IVI (Total)**				
Mean (SD)	75.9 (12.5)	75.2 (11.1)	76.3 (13.3)	0.7
Median (Range)	82 (13–82)	82 (39–82)	82 (13–82)	
**Gender**				
Male				
Mean (SD)	73.8 (12.4)	69 (12.0)	76.9 (12.2)	0.17
Median (Range)	82 (45–82)	68 (52–82)	82 (45–82)	
N	20	8	12	
Female				
Mean (SD)	76.6 (12.5)	77.4 (10.1)	76.1 (13.8)	0.7
Median (Range)	82 (13–82)	82 (39–82)	82 (13–82)	
N	58	22	36	
**Age**				
≤50 years				
Mean (SD)	74.4 (12.5)	74.3 (7.09)	74.5 (15.1)	0.99
Median (Range)	81 (44–82)	73 (68–82)	81.5 (44–82)	
N	9	3	6	
>50 years				
Mean (SD)	76.0 (12.5)	75.3 (11.6)	76.5 (13.2)	0.68
Median (Range)	82 (13–82)	82 (39–82)	82 (13–82)	
N	69	27	42	
**IVI Q1 In the past month, how much has your eyesight interfered with your ability to see and enjoy TV?**				
A lot, a fair amount, a little of the time	12	6	6	0.52
Not at all	65	24	41	
Don’t do this for other reasons	1	0	1	
**IVI Q2 In the past month, how much has your eyesight interfered with taking part in recreational activities such as bowling, walking or golf?**				
A lot, a fair amount, a little of the time	5	1	4	0.65
Not at all	70	27	43	
Don’t do this for other reasons	3	2	1	
**IVI Q3 In the past month, how much has your eyesight interfered with shopping (finding what you want and paying for it)?**				
A lot, a fair amount, a little of the time	10	3	7	0.73
Not at all	67	26	41	
Don’t do this for other reasons	1	1	0	
**IVI Q4 In the past month, how much has your eyesight interfered with visiting friends or family?**				
A lot, a fair amount, a little of the time	6	2	4	1
Not at all	71	27	44	
Don’t do this for other reasons	1	1	0	
**IVI Q5 In the past month, how much has your eyesight interfered with recognising or meeting people?**				
A lot, a fair amount, a little of the time	7	5	2	0.1
Not at all	71	25	46	
Don’t do this for other reasons	0	0	0	
**IVI Q6 In the past month, how much has your eyesight interfered with generally looking after your appearance (face, hair, clothing, etc.)?**				
A lot, a fair amount, a little of the time	4	1	3	1
Not at all	74	29	45	
Don’t do this for other reasons	0	0	0	
**IVI Q7 In the past month, how much has your eyesight interfered with opening packaging (for example, around food, medicines)?**				
A lot, a fair amount, a little of the time	4	1	3	1
Not at all	74	29	45	
Don’t do this for other reasons	0	0	0	
**IVI Q8 In the past month, how much has your eyesight interfered with reading labels or instructions on medicines?**				
A lot, a fair amount, a little of the time	10	3	7	0.73
Not at all	68	27	41	
Don’t do this for other reasons	0	0	0	
**IVI Q9 In the past month, how much has your eyesight interfered with operating household appliances and the telephone?**				
A lot, a fair amount, a little of the time	4	0	4	0.16
Not at all	74	30	44	
Don’t do this for other reasons	0	0	0	
**IVI Q10 How much has your eyesight interfered with moving about outdoors (on the pavement or crossing the street)?**				
A lot, a fair amount, a little of the time	8	3	5	1
Not at all	70	27	43	
Don’t do this for other reasons	0	0	0	
**IVI Q11 In the past month, how much has your eyesight made you move carefully to avoid falling or tripping?**				
A lot, a fair amount, a little of the time	10	4	6	1
Not at all	66	25	41	
Don’t do this for other reasons	2	1	1	
**IVI Q12 In general, how much has your eyesight interfered with travelling or using transport (bus and train)?**				
A lot, a fair amount, a little of the time	10	6	4	0.18
Not at all	67	24	43	
Don’t do this for other reasons	1	0	1	
**IVI Q13 In the past month, how much has your eyesight interfered with going down steps, stairs or curbs?**				
A lot, a fair amount, a little of the time	13	8	5	0.12
Not at all	65	22	43	
Don’t do this for other reasons	0	0	0	
**IVI Q14 In the past month, how much has your eyesight interfered with reading ordinary size print (for example, newspapers)?**				
A lot, a fair amount	13	4	9	0.76
Not at all	64	26	38	
Don’t do this for other reasons	1	0	1	
**IVI Q15 In the past month, how much has your eyesight interfered with getting information that you need?**				
A lot, a fair amount	12	5	7	1
Not at all	66	25	41	
Don’t do this for other reasons	0	0	0	
**IVI Q16 In the past month, how much has your eyesight made you concerned or worried about your general safety at home?**				
A lot, a fair amount, a little of the time	6	3	3	0.67
Not at all	72	27	45	
**IVI Q17 In the past month, how much has your eyesight made you concerned or worried about spilling or breaking things?**				
A lot, a fair amount, a little of the time	4	1	3	1
Not at all	74	29	45	
**IVI Q18 In the past month, how much has your eyesight made you concerned or worried about your general safety when out of your home?**				
A lot, a fair amount, a little of the time	8	5	3	0.25
Not at all	70	25	45	
**IVI Q19 In the past month, how often has your eyesight stopped you from doing the things you want to do?**				
A lot, a fair amount, a little of the time	12	6	6	0.52
Not at all	66	24	42	
**IVI Q20 In the past month, how often have you needed help from other people because of your eyesight?**				
A lot, a fair amount, a little of the time	8	5	3	0.25
Not at all	70	25	45	
**IVI Q21 Have you felt embarrassed because of your eyesight?**				
A lot, a fair amount, a little of the time	5	3	2	0.37
Not at all	73	27	46	
**IVI Q22 Have you felt frustrated or annoyed because of your eyesight?**				
A lot, a fair amount, a little of the time	17	6	11	1
Not at all	61	24	37	
**IVI Q23 Have you felt lonely or isolated because of your eyesight?**				
A lot, a fair amount, a little of the time	4	2	2	0.64
Not at all	74	28	46	
**IVI Q24 Have you ever felt sad or low because of your eyesight?**				
A lot, a fair amount, a little of the time	11	6	5	0.32
Not at all	67	24	43	
**IVI Q25 In the past month, how often have you worried about your eyesight getting worse?**				
A lot, a fair amount, a little of the time	22	10	12	0.45
Not at all	56	20	36	
**IVI Q26 In the past month, how often has your eyesight made you concerned or worried about coping with everyday life?**				
A lot, a fair amount, a little of the time	17	10	7	0.089
Not at all	61	20	41	
**IVI Q27 Have you felt like a nuisance or a burden because of your eyesight?**				
A lot, a fair amount, a little of the time	8	4	4	0.48
Not at all	70	26	44	
**IVI Q28 In the past month, how much has your eyesight interfered with your life in general?**				
A lot, a fair amount, a little of the time	17	10	7	0.089
Not at all	61	20	41	

**Table 9:** Multiple logistic regression with Q26, “How often has your eyesight made you concerned or worried about coping with everyday life?”, as the dependent variable.

Parameter	Model 1 ^†^ Odds Ratio (95% Confidence Interval)	Model 2 ^††^ Odds Ratio (95% Confidence Interval)	Model 3 ^†††^ Odds Ratio (95% Confidence Interval)
Dry eye status	3.25 (1.07, 9.91) *	3.79 (1.19, 12.07) *	3.34 (1.02, 10.95) *

* *p*-value < 0.05; ^†^ Adjusted for dry eye status (0 = Mild DED group, 1 = Severe DED group); ^††^ Adjusted for dry eye status, age and gender; ^†††^ Adjusted for dry eye status, age, gender and FVA (continuous). DED: dry eye disease. FVA: functional visual acuity.

**Table 10:** Multiple logistic regression with Q28, “How much has your eyesight interfered with your life in general?”, as the dependent variable.

Parameter	Model 1 ^†^ Odds Ratio (95% Confidence Interval)	Model 2 ^††^ Odds Ratio (95% Confidence Interval)	Model 3 ^†††^ Odds Ratio (95% Confidence Interval)
Dry eye status	3.25 (1.07, 9.91) *	4.12 (1.23, 13.84) *	3.84 (1.12, 13.2) *

* *p*-value < 0.05; ^†^ Adjusted for dry eye status (0 = Mild DED group, 1 = Severe DED group); ^††^ Adjusted for dry eye status, age and gender; ^†††^ Adjusted for dry eye status, age, gender and FVA (continuous). DED: dry eye disease. FVA: functional visual acuity.

**Table 11:** Functional visual acuity (FVA) association with impact of vision impairment (IVI) questionnaire.

	Overall (*n* = 76)	FVA ≤ 0.6 (*n* = 32)	FVA > 0.6 (*n* = 44)	*p*-Value
**IVI (Total)**				
Mean (SD)	75.7 (12.6)	73.8 (15.9)	77.0 (9.43)	0.28
Median (Range)	82 (13–82)	82 (13–82)	82 (44–82)	
**IVI Q1 In the past month, how much has your eyesight interfered with your ability to see and enjoy TV?**				
A lot, a fair amount, a little of the time	12	4	8	0.54
Not at all	63	28	35	
Don’t do this for other reasons	1	0	1	
**IVI Q2 In the past month, how much has your eyesight interfered with taking part in recreational activities such as bowling, walking or golf?**				
A lot, a fair amount, a little of the time	5	2	3	1
Not at all	68	27	41	
Don’t do this for other reasons	3	3	0	
**IVI Q3 In the past month, how much has your eyesight interfered with shopping (finding what you want and paying for it)?**				
A lot, a fair amount, a little of the time	10	4	6	1
Not at all	65	27	38	
Don’t do this for other reasons	1	1	0	
**IVI Q4 In the past month, how much has your eyesight interfered with visiting friends or family?**				
A lot, a fair amount, a little of the time	6	4	2	0.39
Not at all	69	28	41	
Don’t do this for other reasons	1	0	1	
**IVI Q5 In the past month, how much has your eyesight interfered with recognising or meeting people?**				
A lot, a fair amount, a little of the time	7	4	3	0.45
Not at all	69	28	41	
Don’t do this for other reasons	0	0	0	
**IVI Q6 In the past month, how much has your eyesight interfered with generally looking after your appearance (face, hair, clothing, etc.)?**				
A lot, a fair amount, a little of the time	4	1	3	0.63
Not at all	72	31	41	
Don’t do this for other reasons	0	0	0	
**IVI Q7 In the past month, how much has your eyesight interfered with opening packaging (for example, around food, medicines)?**				
A lot, a fair amount, a little of the time	4	2	2	1
Not at all	72	30	42	
Don’t do this for other reasons	0	0	0	
**IVI Q8 In the past month, how much has your eyesight interfered with reading labels or instructions on medicines?**				
A lot, a fair amount, a little of the time	10	2	8	0.18
Not at all	66	30	36	
Don’t do this for other reasons	0	0	0	
**IVI Q9 In the past month, how much has your eyesight interfered with operating household appliances and the telephone?**				
A lot, a fair amount, a little of the time	4	2	2	1
Not at all	72	30	42	
Don’t do this for other reasons	0	0	0	
**IVI Q10 How much has your eyesight interfered with moving about outdoors (on the pavement or crossing the street)?**				
A lot, a fair amount, a little of the time	8	3	5	1
Not at all	68	29	39	
Don’t do this for other reasons	0	0	0	
**IVI Q11 In the past month, how much has your eyesight made you move carefully to avoid falling or tripping?**				
A lot, a fair amount, a little of the time	10	5	5	0.51
Not at all	64	25	39	
Don’t do this for other reasons	2	2	0	
**IVI Q12 In general, how much has your eyesight interfered with travelling or using transport (bus and train)?**				
A lot, a fair amount, a little of the time	10	6	4	0.3
Not at all	65	25	40	
Don’t do this for other reasons	1	1	0	
**IVI Q13 In the past month, how much has your eyesight interfered with going down steps, stairs or curbs?**				
A lot, a fair amount, a little of the time	13	7	6	0.37
Not at all	63	25	38	
Don’t do this for other reasons	0	0	0	
**IVI Q14 In the past month, how much has your eyesight interfered with reading ordinary size print (for example, newspapers)?**				
A lot, a fair amount	13	5	8	1
Not at all	62	26	36	
Don’t do this for other reasons	1	1	0	
**IVI Q15 In the past month, how much has your eyesight interfered with getting information that you need?**				
A lot, a fair amount	12	4	8	0.55
Not at all	64	28	36	
Don’t do this for other reasons	0	0	0	
**IVI Q16 In the past month, how much has your eyesight made you concerned or worried about your general safety at home?**				
A lot, a fair amount, a little of the time	6	3	3	0.69
Not at all	70	29	41	
**IVI Q17 In the past month, how much has your eyesight made you concerned or worried about spilling or breaking things?**				
A lot, a fair amount, a little of the time	4	3	1	0.3
Not at all	72	29	43	
**IVI Q18 In the past month, how much has your eyesight made you concerned or worried about your general safety when out of your home?**				
A lot, a fair amount, a little of the time	8	4	4	0.71
Not at all	68	28	40	
**IVI Q19 In the past month, how often has your eyesight stopped you from doing the things you want to do?**				
A lot, a fair amount, a little of the time	12	7	5	0.34
Not at all	64	25	39	
**IVI Q20 In the past month, how often have you needed help from other people because of your eyesight?**				
A lot, a fair amount, a little of the time	8	5	3	0.27
Not at all	68	27	41	
**IVI Q21 Have you felt embarrassed because of your eyesight?**				
A lot, a fair amount, a little of the time	5	3	2	0.64
Not at all	71	29	42	
**IVI Q22 Have you felt frustrated or annoyed because of your eyesight?**				
A lot, a fair amount, a little of the time	17	7	10	1
Not at all	59	25	34	
**IVI Q23 Have you felt lonely or isolated because of your eyesight?**				
A lot, a fair amount, a little of the time	4	2	2	1
Not at all	72	30	42	
**IVI Q24 Have you ever felt sad or low because of your eyesight?**				
A lot, a fair amount, a little of the time	11	5	6	1
Not at all	65	27	38	
**IVI Q25 In the past month, how often have you worried about your eyesight getting worse?**				
A lot, a fair amount, a little of the time	22	9	13	1
Not at all	54	23	31	
**IVI Q26 In the past month, how often has your eyesight made you concerned or worried about coping with everyday life?**				
A lot, a fair amount, a little of the time	17	7	10	1
Not at all	59	25	34	
**IVI Q27 Have you felt like a nuisance or a burden because of your eyesight?**				
A lot, a fair amount, a little of the time	8	5	3	0.27
Not at all	68	27	41	
**IVI Q28 In the past month, how much has your eyesight interfered with your life in general?**				
A lot, a fair amount, a little of the time	17	6	11	0.59
Not at all	59	26	33	

## Data Availability

The data presented in this study may be available on request from the correspondence author.
